# Comparative Salivary Proteome of Hepatitis B- and C-Infected Patients

**DOI:** 10.1371/journal.pone.0113683

**Published:** 2014-11-25

**Authors:** Lorena Da Rós Gonçalves, Isabele Batista Campanhon, Romênia R. Domingues, Adriana F. Paes Leme, Márcia Regina Soares da Silva

**Affiliations:** 1 Department of Biochemistry, Chemistry Institute, Federal University of Rio de Janeiro, Rio de Janeiro, RJ, Brazil; 2 Brazilian Biosciences National Laboratory, LNBio, Campinas, SP, Brazil; Saint Louis University, United States of America

## Abstract

Hepatitis B and C virus (HBV and HCV) infections are an important cause of cirrhosis and hepatocellular carcinoma. The natural history has a prominent latent phase, and infected patients may remain undiagnosed; this situation may lead to the continuing spread of these infections in the community. Compelling reasons exist for using saliva as a diagnostic fluid because it meets the demands of being an inexpensive, noninvasive and easy-to-use diagnostic method. Indeed, comparative analysis of the salivary proteome using mass spectrometry is a promising new strategy for identifying biomarkers. Our goal is to apply an Orbitrap-based quantitative approach to explore the salivary proteome profile in HBV- and HCV-infected patients. In the present study, whole saliva was obtained from 20 healthy, (control) 20 HBV-infected and 20 HCV-infected subjects. Two distinct pools containing saliva from 10 subjects of each group were obtained. The samples were ultracentrifuged and fractionated, and all fractions were hydrolyzed (trypsin) and injected into an LTQ-VELOS ORBITRAP. The identification and analyses of peptides were performed using Proteome Discoverer1.3 and ScaffoldQ + v.3.3.1. From a total of 362 distinct proteins identified, 344 proteins were identified in the HBV, 326 in the HCV and 303 in the control groups. Some blood proteins, such as flavin reductase (which converts biliverdin to bilirubin), were detected only in the HCV group. The data showed a reduced presence of complement C3, ceruloplasmin, alpha(1)-acid glycoprotein and alpha(2)-acid glycoprotein in the hepatitis-infected patients. Peptides of serotransferrin and haptoglobin were less detected in the HCV group. This study provides an integrated perspective of the salivary proteome, which should be further explored in future studies targeting specific disease markers for HBV and HCV infection.

## Introduction

Hepatitis B and C are both potentially life-threatening liver infections caused by the hepatitis B and C viruses (HBV and HCV, respectively) and are important global health problems. These conditions can lead to chronic liver disease and put people at high risk of death from cirrhosis of the liver and liver cancer [1], [2]. More than 240 million people have chronic (long-term) liver infections, and approximately 600,000 people die every year due to the acute or chronic consequences of hepatitis B and C. Different from hepatitis B, no vaccine against HCV is available [3].

The natural history of hepatitis B and C virus infections has a prominent latent phase, during which the patient is infected but does not manifest the disease. Infected patients may remain undiagnosed due to an unwillingness of the patient to provide a blood sample for testing. In such situations, HBV and HCV may continue to spread in the community. Therefore, it is of paramount importance to any health care system to detect latently infected individuals in an effort to prevent spreading the infection. This problem can be circumvented by the use of alternative specimens such as saliva for the detection of HCV and HBV infections [4].

Saliva is the secretion of the salivary glands that ensures stability in the oral cavity environment. The oral fluid is composed of saliva itself, gingival crevicular fluids contained in the dentogingival sulcus, mucosal transudate, cell detritus, bacteria and food remains. The basis of saliva is interstitial fluid from blood capillaries that enters via the salivary gland ducts where it is modified from an isotonic to a hypotonic fluid [5].

Saliva has the potential to replace screening using serum/plasma in community-based seroprevalence studies. In general, whole saliva is most frequently studied because its collection is easy, noninvasive and rapid to obtain without the need for specialized equipment. Human saliva harbors proteins of clinical relevance, and approximately 30% of blood proteins are also present in saliva [6], [7]. The discovery of salivary biomarkers and the ongoing development of diagnostic technologies have addressed its diagnostic value for clinical applications [8]. Indeed, human saliva proteomics have proven to be a novel approach in the search for protein biomarkers for the detection of diseases. As a diagnostic specimen in the clinic, saliva has many advantages in terms of collection, storage, shipping and voluminous sampling: all of these processes can be carried out very economically compared with serum or urine. Saliva is also easier to handle during diagnostic procedures than blood because it does not clot, thus reducing the number of manipulations required. For the patient, the noninvasive collection approach could dramatically reduce anxiety and discomfort and increase willingness to undergo health inspections [9].

However, a major barrier to using saliva as a diagnostic fluid has been the fact that many informative analytes are generally present in lower amounts in saliva than in serum. Nonetheless, saliva-based diagnostics may offer a robust alternative for clinicians in the near future when making clinical decisions and predicting post-treatment outcomes [10]. The performance of commercially available immunoassays has been evaluated for anti-HCV antibody screening in saliva. The OraQuick HCV Rapid Antibody Test was reported to have the best performance using these samples but was not better than serum samples, which was previously observed by Chau et al. [11]. This finding may be explained by the low concentration of anti-HCV antibodies present in oral fluid compared with blood or serum, which has been observed using anti-HCV rapid tests and immunoassays with oral fluid samples [12].

The recent availability of mass-spectrometry techniques has improved research on the salivary proteome and has produced qualitative and quantitative information on the protein composition of saliva. As proteomic technologies continue to mature, salivary proteomics can enhance the sensitivity and specificity of human disease detection and have great potential for biomarker research and clinical applications. Salivary proteomic studies are progressively resulting in a growing number of clinical applications for monitoring local and systemic human diseases or conditions such as cardiovascular disease [13], hyperglycemia and diabetes [14] and breast [15] and oral cancers [16]. Nonetheless, there are no results for hepatitis virus infection. Therefore, in this study, human saliva samples were collected from HBV- and HCV-infected patients and matched healthy control subjects. Salivary proteins were identified and compared in two pooled samples from each group by LTQ-VELOS ORBITRAP.

## Materials and Methods

### 2.1. Patient selection

This study was conducted according to the principles expressed in the Declaration of Helsinki. To participate in this study, individuals signed informed consent to a research protocol that had been reviewed and approved by the Federal University of Rio de Janeiro Ethics Committee. All study subjects were a part of a public hepatitis program (Linhares, Espírito Santo – Brazil), and the controls were systemically healthy. Subjects were excluded from the study if they were nursing or pregnant, diabetic or drank alcohol. Each pooled group (HBV- and HCV-infected patients and controls) consisted of 10 individuals (5 female and 5 male). Two pooled samples of each group were collected (biological replicate).

### 2.2 Sample collection and processing

Prior to saliva collection, the volunteers were asked to rest for 15 min, sitting in an upright position, and were asked not to speak until the saliva collection was complete. Unstimulated whole-saliva samples were immediately placed on ice and then centrifuged at 14,000×g for 15 min at 4°C to remove insoluble materials, cell debris and food remnants. The supernatant of each sample was collected, and 1 mM PMSF (Sigma, St. Louis, MO, USA) was added to inhibit proteases. The protein concentration was determined according to the Bradford protein assay. The supernatants were frozen at −70°C until analysis.

### 2.3 Enzymatic digestion for LTQ-Orbritrap experiments and whole-saliva peptide ultrafiltrate preparations

**Figure 1:**
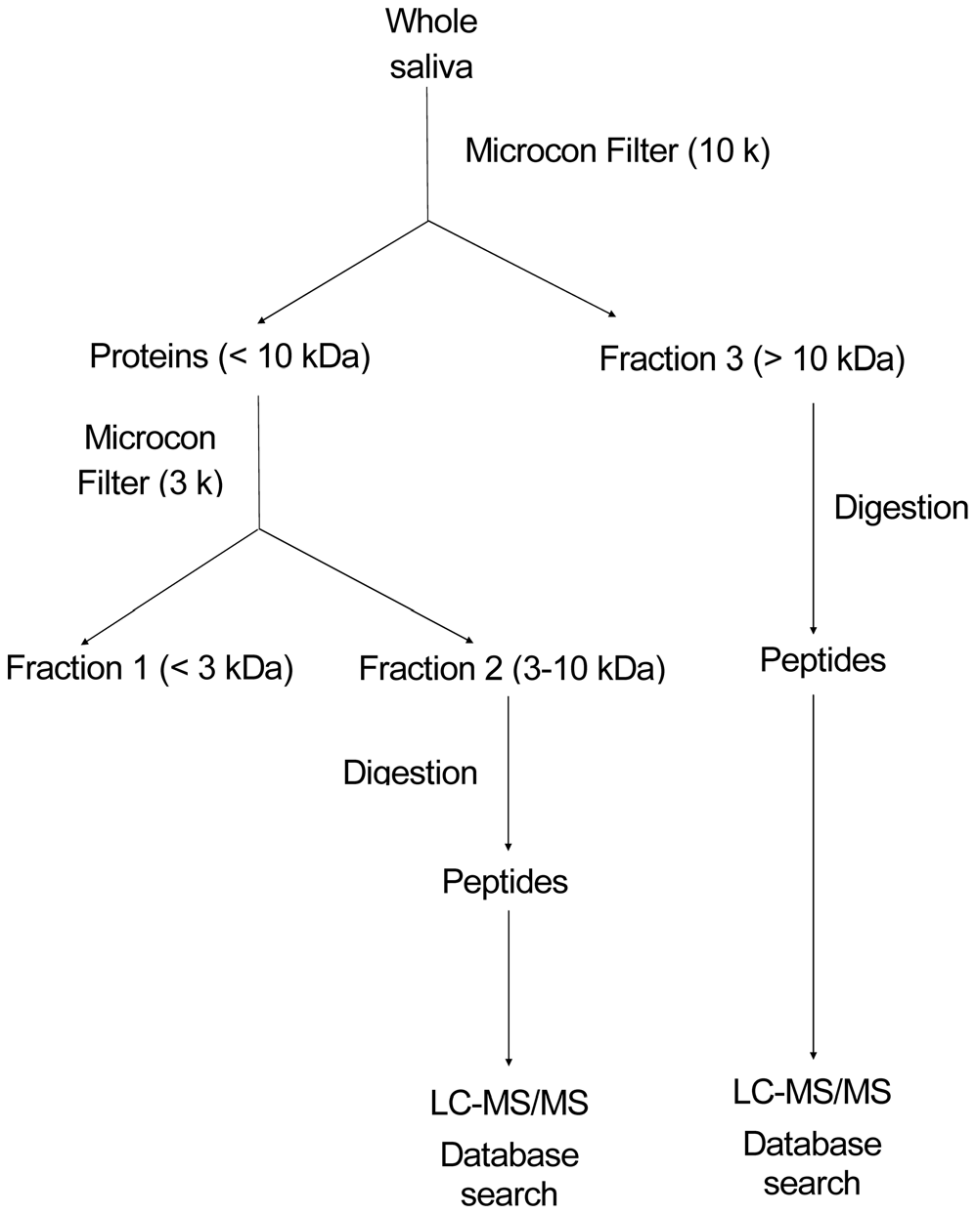
Outline of the procedures for the identification of whole-saliva proteins by prefractionation and shotgun proteomics. Two Microcon ultracentrifuge filters, YM-10K and YM-3K, were used to prefractionate whole saliva into three fractions. Fraction 2 contained proteins within 3–10 kDa, and fraction 3 contained proteins above 10 kDa. The proteins were digested into peptides with trypsin for subsequent protein identification by LCMS/MS and database searching. All steps were performed in duplicate with two biological samples.

The extraction of proteins was performed according to Gonçalves et al. [17]. An aliquot of pooled whole saliva (1 mg/mL) from each group was ultrafiltered through two types of Microcon concentrators (YM-10K and YM-3K, Millipore, Billerica, MA, USA) to separate the whole saliva into three fractions (fraction 1, salivary peptides <3 kDa; fraction 2, enriched for proteins and peptides between 3 and 10 kDa; fraction 3, enriched for proteins >10 kDa) (Figure 1). From 1 mL of whole saliva, approximately 500 µL of fraction 1, 100 µL of fraction 2 and 100 µL of fraction 3 were obtained. All fractions were treated with 10 mM DTT for 30 min and then with 50 mM iodoacetamide for 30 min in the dark. Trypsin (Promega, Madison, WI, USA) (1∶50, w/w) was then added to the sample for overnight tryptic digestion at 37°C. All samples were concentrated in a Speed-Vac centrifuge to produce a 20-µL final volume. Before the experiment, the samples were vacuum-dried and reconstituted with 0.1% formic acid.

### 2.4 Nanoflow liquid chromatography coupled with LTQ Velos Orbitrap

An aliquot containing 4.5 µL of each pool of saliva sample was loaded onto an LTQ Velos Orbitrap mass spectrometer (Thermo Fisher Scientific, Waltham, MA, USA) connected to a nanoflow LC (nLC-MS/MS) by an EASY-nLC system (Proxeon Biosystems, West Palm Beach, FL, USA) through a Proxeon nanoelectrospray ion source. The peptides were separated by a 2%–90% acetonitrile (ACN) gradient in 0.1% formic acid using an analytical column PicoFrit Column (20 cm × ID75 mm, 5 mm particle size, New Objective, Woburn, MA) at a flow of 300 nL/min over 45 min. The nanoelectrospray voltage was set to 1.7 kV, and the source temperature was 275°C. All instrument methods for the LTQ-Orbitrap Velos were set up in the data-dependent acquisition mode. Full-scan MS spectra (m/z 300–1,600) were acquired by the Orbitrap analyzer after accumulation to a target value of 1e6. The resolution of the Orbitrap was set to r = 60,000, and the 20 most intense peptide ions with charge states ≥2 were sequentially isolated to a target value of 5,000 and fragmented in the linear ion trap by low-energy collision-induced dissociation (CID) as the dissociation or fragmentation method, with a normalized collision energy of 35%. The signal threshold for triggering an MS/MS event was set to 500 counts. Dynamic exclusion was enabled with an exclusion size list of 500, an exclusion duration of 60 s and a repeat count of 1. An activation q = 0.25 and an activation time of 10 ms were used. The samples were analyzed in duplicate (technical replicate).

### 2.5 Database searching

Peak lists (msf) were generated from the raw data files using Proteome Discoverer version 1.3 (Thermo Fisher Scientific) with the Sequest search engine and searched against Human International Protein Database (200,740 sequences and 86,640,852 residues) downloaded from UniProt (http://www.uniprot.org) in September 2012. The following parameters were used: carbamidomethylation (+57.021 Da) as a fixed modification; oxidation of methionine (+15.995 Da); phosphorylation of serine, threonine and tyrosine (+79.966 Da) as variable modifications; one trypsin missed cleavage and a tolerance of 10 ppm for precursor and 1 Da for fragment ions. For protein quantification, the data files were analyzed in Scaffold Q+ (version3.3.1, Proteome Software, Inc., Portland, OR, USA); the quantitative value (normalized spectral counts) was obtained with the protein thresholds established at a minimum 90.0% probability and at least 1 peptide with a threshold set to a minimum 60.0% probability and filtered using XCorr cutoffs (+1>1.8, +2>2.2, +3>2.5 and +4>3.5) to have less than 1% FDR. Only peptides with a minimum of five amino acid residues showing a significant threshold (p<0.05) in the Sequest-based score were considered as a product of peptide cleavage. The peptide was considered to be unique when it differed by at least 1 amino acid residue; covalently modified peptides, including N- or C-terminal elongation (i.e., missed cleavages), were counted as unique, and different charge states of the same peptide and modifications were not counted as unique. For the label-free quantization of endogenous peptides, the spectral count and number of unique peptides were assessed. The fold-change between proteins in the HBV, HCV and control groups was also calculated, and the resulting spectrum count values were used to analyze the distribution of identified proteins across the samples.

## Results and Discussion

### 3.1. Human saliva proteomic profiles of hepatitis B- and C-infected patients

The salivary proteomes of hepatitis B (HB)- and hepatitis C (HC)-infected patients were analyzed by liquid chromatography with tandem mass spectrometry (LC-MS/MS). Proteins were identified by the Sequest algorithm against the IPI human protein database and validated using Scaffold software by peptide hits of one or greater. We employed a prefractionation strategy to reduce high-abundance proteins from the samples prior to LC-MS/MS and to improve the representation of low-abundance proteins. Peptide mixtures generated from each sample (digested ultrafiltered pool of saliva – fractions >10 kDa and 3–10 kDa) were analyzed in duplicate, and the cumulative results were derived from the data combination of all 12 samples (two fractions of each salivary pool of the three groups – HBV, HCV and control – and two different pools of each group).

Pooling samples is a common way to reduce the cost of experiments as well as to provide equivalent power to experiments [18]. Because the purpose of our study is to identify robust biomarkers related to the presence HBV and HCV infections, the differences among the groups are more interesting than the differences between the patients within each patient group. We pooled the samples to smooth the intrinsic individual differences and enhance the common characteristic traits only related to the disease. It is also true that pooling samples may eliminate the number of biological replicates.

**Figure 2:**
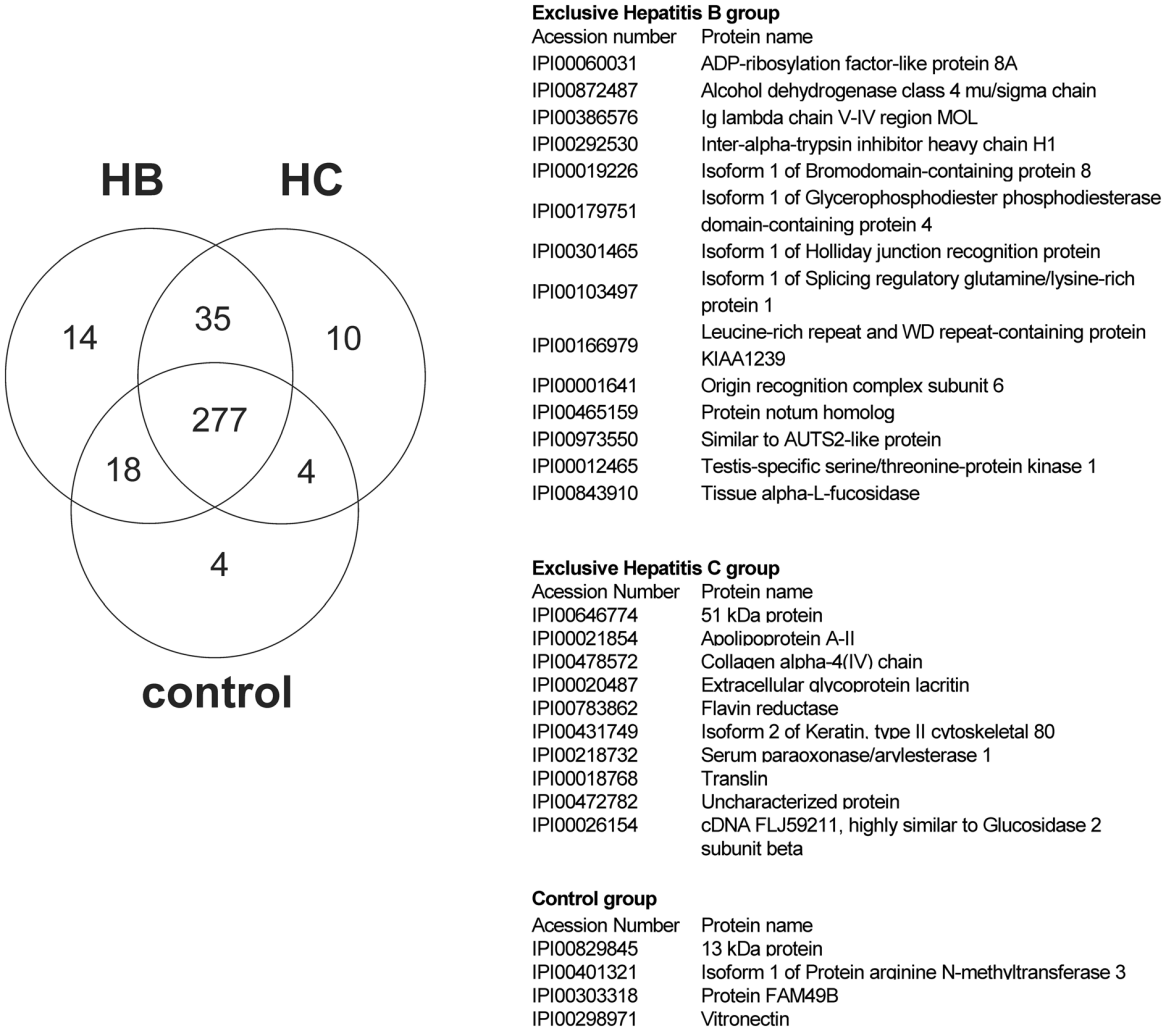
Venn diagram of proteins identified by LC-MS/MS exclusive to patients infected with hepatitis viruses B and C (HBV and HCV) versus the control group and those found in all these groups. The tables list proteins exclusive to the HBV, HCV and control groups.

A consortium of three research groups has catalogued the proteins in human saliva collected as ductal secretions, with 1166 identifications: 914 in parotid and 917 in submandibular/sublingual saliva. The different analysis strategies showed that a high proportion of proteins found in plasma and/or tears are also present in saliva, along with a unique component [19]. Considering the convenience of our gel-free analysis, our study identified 362 proteins, and their distributions are displayed in the Venn diagram in Figure 2. All the human proteins identified in this study are detailed in Table S1. Approximately 273,636 MS/MS peptide spectra for the control group, 226,542 for the HBV group and 222,332 MS/MS for the HCV group were collected (fractions 2 and 3). A total of 344 proteins in the HBV, 326 proteins in the HCV and 303 in the control groups were identified. Of the total proteins identified, 14 were exclusive to the HBV samples, whereas 10 were exclusive to HCV. The control group presented only 4 exclusive proteins. The tables in Figure 2 show the list of proteins exclusive to the HBV, HCV and control groups.

### 3.2. Unique proteins of hepatitis B- and C-infected patients

With regard to HCV infection, one of the specific proteins identified was flavin reductase (IPI IPI00783862), an oxidoreductase that catalyzes NADPH-dependent reduction and in the liver, converts biliverdin to bilirubin. Interestingly, however, both HCV and HBV cause hepatitis, yet HCV appears to be particularly more potent at inducing oxidative stress, suggesting mechanisms that are unique to HCV. Patients with HCV may experience increased oxidative stress due to the activation of NADPH oxidase, increased production of mitochondrial reactive oxygen species (ROS)/reactive nitrogen species (RNS), decreased antioxidants, iron overload and increased cytokines [20], [21]. Clinically, the serum of HCV patients may show increased ROS [22] and RNS, which may lead to the chronic inflammation observed in these patients [23].

### 3.3 Comparative protein analysis of hepatitis B- and C-infected patients

Table 1 lists some of the identified proteins that were selected based on the overall increase or decrease in expression between the groups. As expected, the majority of the detected peptides in all of the fractions correspond to alpha-amylase or serum albumin. The diseased groups showed a reduced number of MS/MS spectra for complement C3, alpha(1)-acid glycoprotein and alpha(2)-acid glycoprotein, haptoglobin, serotransferrin and ceruloplasmin. For simplicity, these differences found in our study are discussed below in sections.

**Table 1:** Spectrum counts of 12 representative proteins present in the saliva of HBV- and HCV-infected patients and identified using the LTQ-VELOS orbitrap.

Accession number	Protein name	Biological function	Spectrum counts		
			HBV-infected patients	HCV-infected patients	Control
IPI00300786	Alpha-amylase 1	Carbohydrate metabolism	2436	1608	1800
IPI00745872	Isoform of Serum albumin	Blood coagulation	942	512	1216
IPI00783987	Complement C3 (Fragment)	activation of the complement system	24	8	40
IPI00022429	Alpha-1-acid-glycoprotein	Acute phase and transport	8	0	24
IPI00020091	Alpha-1-acid-glycoprotein 2	Acute phase and transport	2	2	20
IPI00641737	Haptoglobin	Acute phase and immunity	24	2	36
IPI00465248	Isoform alpha-enolase of alpha-enolase	Glycolysis, Plasminogen activation Transcription, Transcription regulation	78	44	192
IPI00023011	Submaxillary gland androgen-regulated protein 3B	protection or detoxification	20	22	124
IPI00219018	Glyceraldehyde-3-phosphate dehydrogenase	glycolysis and nuclear functions	30	22	80
IPI00032293	Cystatin-C	inhibitor of cysteine proteinases	40	18	28
IPI00022463	Serotransferrin	Iron binding transport	122	76	188
IPI00017601	Cerulopasmin	Transport	8	8	24
IPI00022488	Hemopexin	Binds heme and transports it to the liver for breakdown and iron recovery	28	8	24
IPI00005979	Transthyretin	Transport	4	2	0

#### Complement C3

An interesting finding was the reduced number of MS/MS spectra of complement C3 identified in the saliva of HCV patients (Table 1). The C3 complement protein plays a pivotal role in both the classical and alternative pathways of complement activation. Interactions between HCV and the host immune surveillance system may play an important role in viral persistence. Serum C3 levels have been shown to be depleted in HCV-infected cirrhotic patients [24]. C3 is also an acute-phase protein, the levels of expression of which are either positively or negatively regulated by cytokines during inflammation, chiefly through the regulation of the activities of their cognate genes [25]. Mazumdar et al. [26] suggested that the HCV NS5A protein primarily suppresses C3 complement expression by inhibiting the expression of the IL-1-induced transcription factor C/EBP in human hepatocytes.

#### Alpha(1)-acid glycoprotein and alpha(2)-acid glycoprotein (AAG)

These proteins are acute-phase proteins synthesized predominantly in the liver. Cytokines can cause the plasma AAG level to increase as part of the inflammatory response [27]. The plasma concentration of AAG has been suggested to be a potential marker for cirrhosis and HCC [28], and decreased levels of α(1)-acid glycoprotein are found in patients with chronic hepatitis C [29]. Our study showed a reduced number of MS/MS spectra of these proteins in both groups of hepatitis-infected patients (Table 1).

#### Haptoglobin

This protein is one of the acute-phase proteins secreted by the liver and binds to hemoglobin, playing an important role in the response to inflammation and malignancy. The overall N-glycans of the serum haptoglobin β chain have been found to be different in liver diseases. The Hp β chain contains four potential sites of N-glycosylation, and the site-specific characterization of N-glycans in glycoproteins has a potential clinical application [30]. Sarvary et al. [31] showed that haptoglobin α2 isoforms are differentially expressed in the serum from HCC-patients. Atta et al. [29] reported decreases in the serum levels of α(1)-acid glycoprotein, C3 complement and haptoglobin in HCV-infected patients, similar to our salivary analysis (Table 1). In the HCV group, for example, only two spectra of haptoglobin were detected.

#### Serotransferrin

The quantitative analysis in our study showed that levels of this protein were reduced in the hepatitis-infected patients (Table 1). An estimated 30–40% of patients with chronic hepatitis C have elevated serum iron, transferrin saturation and increased ferritin levels, and clinical data suggest that iron is a co-morbidity factor for disease progression following hepatitis-virus infection. Although iron is essential for a number of fundamental metabolic processes in cells and organisms, mammalian iron homeostasis is tightly regulated through the coordinated action of sensory and regulatory networks that modulate the expression of iron-related proteins at the transcriptional and/or posttranscriptional levels. Disturbances in iron homeostasis have been implicated in infectious disease pathogenesis. Viruses, similar to other pathogens, can escape recognition by the immune system, but they need iron from their host to grow and spread [32]. Increased levels of ferritin and serum iron levels have been correlated with progressive hepatic parenchymal disease. A possible explanation for these elevations is that a necroinflammatory hepatic status can release iron and ferritin from damaged hepatocytes, a process also sustained by the concomitant high serum levels of serum alanine aminotransferase. Furthermore, iron accumulation in HBV and HCV infections [33] causes liver damage due to oxidative stress, which increases hepatocyte necrosis/apoptosis, hepatic stellate cell activation and fibrogenesis through the proliferation of actin and collagen [34], [35]. However, iron markers do not differ significantly in patients with low or high HCV viremia [36]. Serum ferritin and transferrin levels appear to play an important role in determining the severity of liver disease related both to liver fibrosis and necroinflammatory activity but not the presence of infection.

#### Ceruloplasmin

Ceruloplasmin is an alpha 2-glycoprotein that is mainly synthesized in the liver and has been shown to play a role in acute-phase reactions, in which serum ceruloplasmin levels are upregulated during inflammation and/or tissue damage. Although the levels of this protein are downregulated in conditions such as severe hepatitis, fulminant hepatitis and decompensated cirrhosis [37]-[39], the clinical significance of ceruloplasmin has not yet been clearly defined [40]. In this study, we showed that peptides of ceruloplasmin were less detected in the HBV- and HCV-infected groups compared to the control group (Table 1).

#### Hemopexin, transthyretin, enolase-1, thrombospondim-1, cystatin C, glyceraldehydes-3-dehydrogenase (GADPH) and submaxillary gland androgen-regulated protein 3B

Proteomics has proven to be useful for elucidating the pathology of and discovering disease markers for hepatocellular carcinoma (HCC). Several studies have employed mass spectrometry techniques to compare sera from HCC patients and their possible correlation with HBV and HCV infections [41], [42]. Some serum HCC biomarker candidates have been suggested, such as hemopexin [43], transthyretin, α-fetoprotein [44], GADPH [45], alpha-enolase and thrombospondim-1 [7], [46] and cystatin C [47]. Except for α-fetoprotein and thrombospondim-1, our study also detected these proteins in saliva samples, but the quantitative alterations were not the same in the presence of hepatitis as detected in the presence of hepatic cancer (Table 1). Submaxillary gland androgen-regulated protein 3B, which has been detected as increased in oral squamous cell carcinoma, was reduced in the hepatitis-infected patients [16].

## Conclusions

Due to its advantages of an easy, safe, cost-effective and non-invasive diagnostic approach, saliva shows a high potential for diagnostic hepatitis virus infections. Considering the fact that a high proportion of proteins found in plasma and/or tears are also present in saliva, human saliva proteomics has proven to be a novel approach in the search for protein biomarkers for the non-invasive detection of human diseases. This is the first study to describe salivary protein alterations associated with hepatitis B and C. These findings may provide novel insight into salivary protein differences between HBV- and HCV-infected patients and help to identify candidate biomarkers that may lead to more efficient hepatitis diagnosis in an epidemiological setting.

## Supporting Information

Table S1 **Complete list of proteins identified in the proteome analysis of control, HBV and HCV-infected patients.**
(PDF)

## References

[1] Poynard T, Yuen MF, Ratziu V, Lai CL. Viral hepatitis C. Lancet. 2003;362:2095–2100. 10.1016/s0140-6736(03)15109-4 14697814

[2] Trépo C, Chan HL, Lok A. Hepatitis B virus infection. 2014; Lancet pii: S0140-6736(14)60220-8. 10.1016/S0140-6736(14)60220-8 24954675

[3] Fan X, Xue B, Dolan PT, LaCount DJ, Kurgan L, et al The intrinsic disorder status of the human hepatitis C virus proteome. Mol Biosyst. 2014;10:1345–63. 24752801 10.1039/c4mb00027g

[4] Villar LM, de Paula VS, de Almeida AJ, Rodrigues do Ó KM, Miguel JC, et al Kowledge and prevalence of viral hepatitis among beauticians. Sep; 86(9): 1515-21J Med Virol 2014;86:1515–21. 10.1002/jmv.23993 24916521

[5] Wong DT. Salivaomics. J Am Dent Assoc. 2012;143:19S–24S. 23034834 10.14219/jada.archive.2012.0339

[6] Loo JA, Yan W, Ramachandran P, Wong DT. Comparative human salivary and plasma proteomes. J Dent Res. 2010;89:1016–23. 20739693 10.1177/0022034510380414 PMC3144065

[7] Zhang A, Sun H, Wang P, Wang X. Salivary proteomics in biomedical research. Clin Chim Acta. 2013;16:261–5. 10.1016/j.cca.2012.11.001 23146870

[8] Castagnola M, Picciotti PM, Messana I, Fanali C, Fiorita A, et al Potential applications of human saliva as diagnostic fluid. Acta Otorhinolaryngol Ital. 2011;31:347–57. 22323845 PMC3272865

[9] Schulz BL, Cooper-White J, Punyadeera CK. Saliva proteome research: current status and future outlook. Crit Rev Biotechnol. 2013;33:246–59. 22612344 10.3109/07388551.2012.687361

[10] Amado FM, Ferreira RP, Vitorino R. One decade of salivary proteomics: current approaches and outstanding challenges. Clin Biochem. 2013;46:506–17. 23103441 10.1016/j.clinbiochem.2012.10.024

[11] Cha YJ, Park Q, Kang ES, Yoo BC, Park KU, et al.. Performance evaluation of the OraQuick hepatitis C virus rapid antibody test. 2013. 10.3343/alm.2013.33.3.184 PMC3646192 23667844

[12] Moorthy M, Daniel HD, Kurian G, Abraham P. An evaluation of saliva as an alternative to plasma for the detection of hepatitis C virus antibodies. Indian J Med Microbiol. 2008;26:327–32. 18974484 10.4103/0255-0857.42116

[13] Miller CS, Foley JD 3rd, Floriano PN, Christodoulides N, Ebersole JL et al. Utility of Salivary Biomarkers for Demonstrating Acute Myocardial Infarction. J Dent Res. 2014;30:72S–79S. 10.1177/0022034514537522 PMC4107546 24879575

[14] Bencharit S, Baxter SS, Carlson J, Byrd WC, Mayo M, et al.. Salivary proteins associated with hyperglycemia in diabetes: a proteomic analysis. 2013. 10.1039/c3mb70196d PMC3888809 24056972

[15] Streckfus C, Bigler L, Dellinger T, Dai X, et al The presence of soluble c-erbB-2 in saliva and serum among women with breast carcinoma: a preliminary study. Clin Cancer Res. 2000;6:2363–2370. 10873088

[16] Koffler J, Holzinger D, Sanhueza GA, Flechtenmacher C, Zaoui K, et al Submaxillary gland androgen-regulated protein 3A expression is an unfavorable risk factor for the survival of oropharyngeal squamous cell carcinoma patients after surgery. Eur Arch Otorhinolaryngol. 2013;270:1493–500. 23053383 10.1007/s00405-012-2201-6

[17] Gonçalves L da R, Soares MR, Nogueira FC, Garcia C, Camisasca DR, et al Comparative proteomic analysis of whole saliva from chronic periodontitis patients. J Proteomics. 2010;73:1334–41. 20215060 10.1016/j.jprot.2010.02.018

[18] Jehmlich N, Dinh KH, Gesell-Salazar M, Hammer E, Steil L, et al Quantitative analysis of the intra- and inter-subject variability of the whole salivary proteome. J Periodontal Res. 2013;48:392–403. 23164135 10.1111/jre.12025

[19] Denny P, Hagen FK, Hardt M, Liao L, Yan W, et al The proteomes of human parotid and submandibular/sublingual gland salivas collected as the ductal secretions. J Proteome Res. 2008;7:1994–2006. 18361515 10.1021/pr700764j PMC2839126

[20] Gong G, Waris G, Tanveer R, Siddiqui A. Human hepatitis C virus NS5A protein alters intracellular calcium levels, induces oxidative stress, and activates STAT-3 and NF-kappa B. Proc Natl Acad Sci U S A. 2001;98:9599–9604. 11481452 10.1073/pnas.171311298 PMC55498

[21] Okuda M, Li K, Beard MR, Showalter LA, Scholle F, Lemon SM, et al Mitochondrial injury, oxidative stress, and antioxidant gene expression are induced by hepatitis C virus core protein. Gastroenterology. 2002;122:366–375. 11832451 10.1053/gast.2002.30983

[22] Choi J, Lee KJ, Zheng Y, Yamaga AK, Lai MM, et al Reactive oxygen species suppress hepatitis C virus RNA replication in human hepatoma cells. Hepatology. 2004;39:81–89. 14752826 10.1002/hep.20001

[23] Choi J, Ou JH. Mechanisms of liver injury. III Oxidative stress in the pathogenesis of hepatitis C virus. Am J Physiol Gastrointest Liver Physiol. 2006;290:G847–851. 16603728 10.1152/ajpgi.00522.2005

[24] Gangadharan B, Antrobus R, Chittenden D, Rossa J, Bapat M, et al New approaches for biomarker discovery: the search for liver fibrosis markers in hepatitis C patients. J Proteome Res. 2011;10:2643–50. 21410221 10.1021/pr101077c PMC3089987

[25] Ricklin D, Hajishengallis G, Yang K, Lambris JD. Complement: akey system for immune surveillance and homeostasis. Nat. Immunol. 2010;11:785–797. 10.1038/ni.1923 PMC2924908 20720586

[26] Mazumdar B, Kim H, Meyer K, Bose SK, Di Bisceglie AM, et al Hepatitis C virus proteins inhibit C3 complement production. J Virol. 2012;86:2221–8. 22171262 10.1128/JVI.06577-11 PMC3302406

[27] Fournier T, Medjoubi N, Porquet D. α-1-acid glycoprotein. Biochim Biophys Acta. 2000;1482:157–171. 11058758 10.1016/s0167-4838(00)00153-9

[28] Bachtiar I, Kheng V, Wibowo GA, Gani RA, Hasan I, et al Alpha-1-acid glycoprotein as potential biomarker for alpha-fetoprotein-low hepatocellular carcinoma. BMC Res Notes. 2010;23:319. 10.1186/1756-0500-3-319 PMC2999612 21092242

[29] Atta M, Cabral M, Santos G, Paraná R, Atta A. Inflammation biomarkers in chronic hepatitis C: association with liver histopathology, HCV genotype and cryoglobulinemia. Inflamm Res. 2012;61:1101–6. 22718074 10.1007/s00011-012-0502-2

[30] Zhang S, Jiang K, Sun C, Lu H, Liu Y. Quantitative analysis of site-specific N-glycans on sera haptoglobin β chain in liver diseases. Acta Biochim Biophys Sin. 2013;45:1021–9. 24103369 10.1093/abbs/gmt110

[31] Sarvari J, Mojtahedi Z, Taghavi SA, Kuramitsu Y, Shamsi Shahrabadi M, et al Differentially Expressed Proteins in Chronic Active Hepatitis, Cirrhosis, and HCC Related to HCV Infection in Comparison With HBV Infection: A proteomics study. Hepat Mon. 2013;13:e8351. 24066001 10.5812/hepatmon.8351 PMC3776151

[32] Georgopoulou U, Dimitriadis A, Foka P, Karamichali E, Mamalaki A. Hepcidin and the iron enigma in HCV infection. Virulence. 2014;15:465–76. 10.4161/viru.28508 PMC4063809 24626108

[33] Price L, Kowdley KV. The role of iron in the pathophysiology and treatment of chronic hepatitis C. Can J Gastroenterol. 2009;23:822–8. 20011735 10.1155/2009/290383 PMC2805519

[34] Ramm GA, Ruddell RG. Hepatotoxicity of iron overload: mechanisms of iron-induced hepatic fibrogenesis. Semin Liver Dis. 2005;25:433–49. 16315137 10.1055/s-2005-923315

[35] Fujita N, Sugimoto R, Ma N, Tanaka H, Iwasa M, et al Comparison of hepatic oxidative DNA damage in patients with chronic hepatitis B and C. J Viral Hepat. 2008;15:498–507. 18331251 10.1111/j.1365-2893.2008.00972.x

[36] Vagu C, Sultana C, Ruta S. Serum iron markers in patients with chronic hepatitis C infection. Hepat Mon. 2013;13:e13136. 24348638 10.5812/hepatmon.13136 PMC3842519

[37] Yang X, Tong DJ, Liang J, Zhang YH, Lei JH, et al Ceruloplasmin level of patients with liver disease in China. ZhonghuaNei Ke Za Zhi. 2005;44:13–15. 15769390

[38] Perman JA, Werlin SL, Grand RJ, Watkins JB. Laboratory measures of copper metabolism in the differentiation of chronic active hepatitis and Wilson disease in children. J Pediatr. 1979;94:564–568. 430291 10.1016/s0022-3476(79)80011-6

[39] Walshe JM. JB Ceruloplasmin in liver disease. Lancet. 1962;vol ii:263–265.

[40] Zeng DW, Liu YR, Zhang JM, Zhu YY, Lin S, et al Serum ceruloplasmin levels correlate negatively with liver fibrosis in males with chronic hepatitis B: a new noninvasive model for predicting liver fibrosis in HBV-related liver disease. PLoS One. 2013;25:e77942. 10.1371/journal.pone.0077942 PMC3837017 24282481

[41] Fye HK, Wright-Drakesmith C, Kramer HB, Camey S, Nogueira da Costa A, et al Protein profiling in hepatocellular carcinoma by label-free quantitative proteomics in two west African populations. PLoS One. 2013;30:e68381. 10.1371/journal.pone.0068381 PMC3728326 23935864

[42] Rath T, Hage L, Kügler M, Menendez Menendez K, Zachoval R, et al Serum proteome profiling identifies novel and powerful markers of cystic fibrosis liver disease. PLoS One. 2013;8:e58955. 23516586 10.1371/journal.pone.0058955 PMC3597583

[43] Kobayashi S, Nouso K, Kinugasa H, Takeuchi Y, Tomoda T, et al Clinical utility of serum fucosylated hemopexin in Japanese patients with hepatocellular carcinoma. Hepatol Res. 2012;42:1187–95. 22639859 10.1111/j.1872-034X.2012.01044.x

[44] Feng JT, Liu YK, Song HY, Dai Z, Qin LX, et al Heat-shock protein 27: a potential biomarker for hepatocellular carcinoma identified by serum proteome analysis. Proteomics. 2005;5:4581–8. 16240287 10.1002/pmic.200401309

[45] Ganapathy-Kanniappan S, Kunjithapatham R, Geschwind JF. Glyceraldehyde-3-phosphate dehydrogenase: a promising target for molecular therapy in hepatocellular carcinoma. Oncotarget. 2012;3:940–53. 22964488 10.18632/oncotarget.623 PMC3660062

[46] Takashima M, Kuramitsu Y, Yokoyama Y, Iizuka N, Fujimoto M, et al Overexpression of alpha enolase in hepatitis C virus-related hepatocellular carcinoma: association with tumor progression as determined by proteomic analysis. Proteomics. 2005;5:1686–92. 15800975 10.1002/pmic.200401022

[47] Zinkin NT, Grall F, Bhaskar K, Otu HH, Spentzos D, et al Serum proteomics and biomarkers in hepatocellular carcinoma and chronic liver disease. Clin Cancer Res. 2008;14:470–7. 18223221 10.1158/1078-0432.CCR-07-0586

