# Supplementary material for: Comparative Salivary Proteome of Hepatitis B- and C-Infected Patients
Source: PLoS One. 2014 Nov 25;9(11):e113683. doi: 10.1371/journal.pone.0113683 (PMC4244100; doi:10.1371/journal.pone.0113683)
Supplement: Table S1 — Complete list of proteins identified in the proteome analysis of control, HBV and HCV-infected patients. (PDF) [file pone.0113683.s001.pdf]

Table S1: Complete list of identified proteins in HB, HC and Control (C) groups.

**Identified Proteins (362)**	**Accession Number**	**Spectrum counts**		
		**HB**	**HC**	**C**
Alpha-amylase 1	IPI00300786	2436	1608	1800
Isoform 1 of Serum albumin	IPI00745872	942	512	1216
Polymeric immunoglobulin receptor	IPI00004573	1066	680	724
Prolactin-inducible protein	IPI00022974	658	452	944
cDNA FLJ14473 fis, clone MAMMA1001080 , highly similar to Homo sapiens SNC73 protein (SNC73) mRNA	IPI00386879	566	326	528
Cystatin-S	IPI00032294	598	484	736
Uncharacterized protein	IPI00855918 (+1)	572	380	524
IGK@ protein	IPI00784985	454	400	404
Cystatin-SN	IPI00305477	434	332	360
Zymogen granule protein 16 homolog B	IPI00060800	264	198	260
cDNA FLJ60163, highly similar to Carbonic anhydrase 6	IPI00295105	184	124	160
Cystatin-SA	IPI00013382	244	212	240
IGL@ protein	IPI00154742	180	80	176
Actin, cytoplasmic 2	IPI00021440	94	62	284
Serotransferrin	IPI00022463	122	76	188
Keratin, type II cytoskeletal 1	IPI00220327	54	162	108
cDNA FLJ36533 fis, clone TRACH2004428, highly similar to Lactotransferrin (Fragment)	IPI00903112	72	110	148
Uncharacterized protein	IPI00925214 (+1)	98	66	36
Lactoperoxidase	IPI00025023	90	58	100
Keratin, type I cytoskeletal 10	IPI00009865	64	122	92
Hemoglobin subunit alpha	IPI00410714	34	164	12
Isoform alpha-enolase of Alpha-enolase	IPI00465248	78	44	192
UPF0762 protein C6orf58	IPI00374315	70	36	112
Bactericidal/permeability-increasing protein-like 1	IPI00296654	82	66	60
Isoform 1 of Deleted in malignant brain tumors 1 protein	IPI00099110 (+5)	68	46	64
Keratin, type I cytoskeletal 9	IPI00019359	46	76	36
Lipocalin-1	IPI00009650	74	46	104
Alpha-2-macroglobulin	IPI00478003 (+1)	68	50	88
Immunoglobulin J chain	IPI00178926 (+1)	80	62	36
Keratin, type II cytoskeletal 2 epidermal	IPI00021304	34	86	48
Titin, isoform CRA_a	IPI00940872	2	0	0
Immunoglobulin heavy chain variant (Fragment)	IPI00940245	64	32	48
Lysozyme C	IPI00019038	32	38	68
Putative uncharacterized protein DKFZp686P15220	IPI00645363	50	30	72
Hemoglobin subunit beta	IPI00654755	36	62	32
Alpha-2-macroglobulin-like protein 1	IPI00419215	46	24	72
Submaxillary gland androgen-regulated protein 3B	IPI00023011	20	22	124
Isoform 2 of Ig mu chain C region	IPI00896380	46	10	44
Plastin-2	IPI00010471	48	22	44
Zinc-alpha-2-glycoprotein	IPI00166729	40	24	56
Transcobalamin-1	IPI00299729	54	26	44
Thioredoxin	IPI00216298	54	34	40
Glyceraldehyde-3-phosphate dehydrogenase	IPI00219018	30	22	80

Table S1: Complete list of identified proteins in HB, HC and Control (C) groups.

hypothetical protein LOC644893	IPI00977368	0	4	12
Protein S100-A8	IPI00007047	50	24	32
Short palate, lung and nasal epithelium carcinoma-associated protein 2	IPI00304557	84	36	24
Glucose-6-phosphate isomerase	IPI00908881	44	20	32
Putative uncharacterized protein DKFZp686O16217 (Fragment)	IPI01015266	58	18	8
Cystatin-C	IPI00032293	40	18	28
Complement C3 (Fragment)	IPI00783987	24	8	40
Isoform 1 of Gelsolin	IPI00026314	28	26	32
Profilin-1	IPI00216691	28	18	72
Protein S100-A9	IPI00027462	28	8	48
Isoform 1 of Alpha-1-antitrypsin	IPI00553177	14	28	20
Isoform 1 of Long palate, lung and nasal epithelium carcinoma-associated protein 1	IPI00291410	36	20	16
Heat shock 70 kDa protein 1A/1B	IPI00304925 (+1)	26	12	56
Glutathione S-transferase P	IPI00219757	40	14	32
Protein disulfide-isomerase	IPI00010796	18	14	20
IgGFc-binding protein	IPI00242956	40	4	20
Isoform 2A of Desmocollin-2	IPI00025846 (+1)	28	30	12
Myosin-reactive immunoglobulin heavy chain variable region (Fragment)	IPI00384392	34	24	24
6-phosphogluconate dehydrogenase, decarboxylating	IPI00219525 (+1)	36	32	48
Peptidyl-prolyl cis-trans isomerase A	IPI00419585	24	12	24
Hemopexin	IPI00022488	28	8	24
Desmoglein-1	IPI00025753	6	18	36
Isoform 1 of Nucleobindin-2	IPI00009123	22	18	24
Galectin-3-binding protein	IPI00023673 (+1)	20	24	12
Matrix metalloproteinase-9	IPI00027509	16	8	28
Isoform H17 of Myeloperoxidase	IPI00007244 (+2)	26	8	36
Cystatin-D	IPI00002851	14	14	36
Small proline-rich protein 3	IPI00082931 (+1)	12	12	12
Isoform 1 of L-lactate dehydrogenase A chain	IPI00217966 (+1)	16	12	32
Fatty acid-binding protein, epidermal	IPI00007797	20	2	24
Phosphoglycerate kinase 1	IPI00169383	14	6	40
Uncharacterized protein	IPI00965085	14	8	28
Desmoglein-3	IPI00031547	26	12	28
cDNA FLJ52432, highly similar to Leukotriene A-4 hydrolase	IPI00790203	26	10	16
Apolipoprotein A-I	IPI00021841	20	8	20
Isoform M2 of Pyruvate kinase isozymes M1/M2	IPI00479186	14	16	24
Leukocyte elastase inhibitor	IPI00027444 (+1)	16	16	16
Haptoglobin	IPI00641737	24	2	36
Cornulin	IPI00297056	20	12	4
Fibrinogen beta chain	IPI00298497	10	18	24
triosephosphate isomerase isoform 2	IPI00465028 (+1)	20	16	16
Keratin, type I cytoskeletal 16	IPI00217963	16	22	28
Keratin, type II cytoskeletal 5	IPI00009867	4	26	20
cDNA FLJ54957, highly similar to Transketolase	IPI00643920 (+1)	18	10	8
Anti-(ED-B) scFV (Fragment)	IPI00916434	24	14	32

Table S1: Complete list of identified proteins in HB, HC and Control (C) groups.

Isoform 1 of Kallikrein-1	IPI00304808	12	8	32
Mucin-7	IPI00152154	6	0	12
Retinoblastoma-like protein 2	IPI00304028	2	0	0
Isoform 1 of 14-3-3 protein sigma	IPI00013890	16	8	24
Cathepsin D	IPI00011229	20	10	24
14-3-3 protein zeta/delta	IPI00021263	22	14	24
Transaldolase	IPI00744692	14	4	20
Cystatin-B	IPI00021828	18	8	16
Phosphoglycerate mutase 1	IPI00549725	30	2	24
Histatin-3	IPI00012026	0	0	4
Isoform 1 of Interleukin-1 receptor antagonist protein	IPI00000045 (+3)	8	4	8
Ceruloplasmin	IPI00017601	8	8	24
Isoform Gamma-B of Fibrinogen gamma chain	IPI00021891 (+3)	10	8	12
45 kDa protein	IPI00796333 (+1)	14	6	24
Isoform Sap-mu-9 of Proactivator polypeptide	IPI00744835	14	6	16
Beta-2-microglobulin	IPI00004656	10	14	8
Isoform 2 of Obscurin	IPI00742748	0	0	4
cDNA FLJ60299, highly similar to Rab GDP dissociation inhibitor beta	IPI00031461 (+1)	16	2	16
Putative uncharacterized protein DKFZp686C15213	IPI00426051	20	8	24
78 kDa glucose-regulated protein	IPI00003362	20	12	16
cDNA FLJ53019, highly similar to Serpin B13	IPI00006560 (+1)	18	4	12
Alpha-actinin-4	IPI00013808 (+1)	14	6	20
Isoform 1 of Heat shock cognate 71 kDa protein	IPI00003865	16	8	24
Sortilin-related receptor	IPI00022608	2	2	4
Moesin	IPI00219365	12	6	16
glycogen phosphorylase, liver form isoform 2	IPI00943894	8	4	28
Rho GDP-dissociation inhibitor 2	IPI00003817	4	6	20
Peroxiredoxin-1	IPI00000874	10	10	8
Peroxiredoxin-6	IPI00220301	8	2	12
cDNA FLJ41552 fis, clone COLON2004478, highly similar to Protein Tro alpha1 H,myeloma	IPI00647704	10	4	4
Isoform 1 of Dipeptidyl peptidase 1	IPI00022810 (+1)	6	6	8
Isoform 1 of WAP four-disulfide core domain protein 2	IPI00291488	18	2	12
cDNA FLJ39690 fis, clone SMINT2010639, highly similar to PHOSPHOLIPID TRANSFER PROTEIN	IPI00643034 (+1)	8	10	4
Calmodulin-like protein 3	IPI00216984	10	8	12
Isoform 1 of Adenylyl cyclase-associated protein 1	IPI00008274 (+1)	4	10	24
Cornifin-A	IPI00017987	10	2	4
Leucine-rich alpha-2-glycoprotein	IPI00022417	8	2	16
Cofilin-1	IPI00012011 (+2)	6	10	16
Isoform 1 of Dynein heavy chain 2, axonemal	IPI00783464	0	2	0
Ly6/PLAUR domain-containing protein 3	IPI00004310 (+1)	16	4	12
Isoform 1 of Clusterin	IPI00291262 (+3)	8	10	8
Peptidyl-prolyl cis-trans isomerase B	IPI00646304	4	6	20
Isoform 1 of Acyl-CoA-binding protein	IPI00010182 (+5)	4	6	4
Rheumatoid factor C6 light chain (Fragment)	IPI00829956	22	4	12

Table S1: Complete list of identified proteins in HB, HC and Control (C) groups.

Similar to VH4 heavy chain variable region precursor	IPI00977297	12	0	0
Ig heavy chain V-III region GAL	IPI00382500	10	4	8
Isoform DPI of Desmoplakin	IPI00013933 (+2)	4	14	8
cDNA FLJ25678 fis, clone TST04067, highly similar to PURINE NUCLEOSIDE PHOSPHORYLASE	IPI00017672	14	4	16
Alpha-1-acid glycoprotein 1	IPI00022429	8	0	24
Isoform 1 of Fibrinogen alpha chain	IPI00021885	2	6	0
Protein S100-A11	IPI00013895	8	4	16
Isoform f-I of Serine protease inhibitor Kazal-type 5	IPI00478816 (+1)	8	4	20
Transgelin-2	IPI00550363 (+2)	14	8	4
Isoform 5 of Peptidyl-glycine alpha-amidating monooxygenase	IPI00177543 (+2)	4	4	0
Nucleobindin-1	IPI00295542	4	6	8
Isoform Long of Glucose-6-phosphate 1-dehydrogenase	IPI00216008 (+3)	6	4	4
Putative uncharacterized protein	IPI00010402 (+2)	10	12	12
Rheumatoid factor D5 light chain (Fragment)	IPI00816799 (+1)	12	8	12
Putative uncharacterized protein DKFZp686M24218	IPI00930442	12	4	8
Isoform 1 of Chromodomain-helicase-DNA-binding protein 1	IPI00297851 (+1)	0	0	4
Malate dehydrogenase, cytoplasmic	IPI00916111 (+1)	10	6	8
Ig kappa chain V-II region TEW	IPI00736885	6	2	8
Isoform 1 of von Willebrand factor A domain-containing protein 3A	IPI00740853	2	2	0
Ig kappa chain V-IV region Len	IPI00387120	12	8	4
Retrotransposon gag domain-containing protein 1	IPI00514462	0	0	4
Sin3 histone deacetylase corepressor complex component SDS3	IPI00607645 (+1)	2	6	0
Protein-glutamine gamma-glutamyltransferase E	IPI00300376	4	4	0
Putative uncharacterized protein	IPI00973474	8	2	8
Ig heavy chain V-III region BUT	IPI00382481	4	2	4
Myosin-reactive immunoglobulin heavy chain variable region (Fragment)	IPI00783024	6	6	12
Uncharacterized protein	IPI00952742	4	0	0
Keratin, type II cytoskeletal 6C	IPI00299145 (+2)	4	4	20
Transmembrane protease serine 11D	IPI00003542 (+1)	12	6	4
Isoform 1 of Hemicentin-2	IPI00936570	0	2	0
cDNA FLJ55606, highly similar to Alpha-2-HS-glycoprotein	IPI00022431 (+1)	8	4	8
cysteine-rich secretory protein 3 isoform 1 precursor	IPI00942117 (+1)	6	0	20
Isoform 1 of Nexilin	IPI00180404 (+3)	2	0	0
suprabasin isoform 1 precursor	IPI00947285	0	6	16
Antileukoproteinase	IPI00008580	4	2	0
Uncharacterized protein	IPI00643525 (+6)	8	2	16
Isoform 1 of Coiled-coil domain-containing protein 112	IPI00746232 (+2)	4	4	0
Trefoil factor 3	IPI00018909	4	2	8
Ig heavy chain V-III region WEA	IPI00382476	6	2	4
Calmodulin	IPI00075248 (+3)	6	8	4
Metalloproteinase inhibitor 1	IPI00032292 (+1)	8	2	12
Histone H4	IPI00453473	12	6	4
Uncharacterized protein	IPI00936857 (+1)	0	4	0
cDNA FLJ35730 fis, clone TESTI2003131, highly similar to ALPHA-1-ANTICHYMOTRYPSIN	IPI00550991 (+1)	8	4	12

Table S1: Complete list of identified proteins in HB, HC and Control (C) groups.

Carboxypeptidase E precursor	IPI00031121 (+1)	4	0	8
Carbonic anhydrase 1	IPI00215983	2	14	0
Catalase	IPI00465436	8	2	8
SPARC-like protein 1	IPI00296777	2	4	12
Monocyte differentiation antigen CD14	IPI00029260 (+1)	6	6	4
Chitinase-3-like protein 2	IPI00019533 (+1)	6	4	4
Ig kappa chain V-I region Roy	IPI00387100	10	6	8
Angiotensinogen	IPI00032220	8	2	20
ERO1-like protein alpha	IPI00386755	2	0	8
Phosphatidylethanolamine-binding protein 1	IPI00219446	16	2	4
Isoform 1 of Kallikrein-11	IPI00002818 (+2)	6	0	8
Calreticulin	IPI00020599	4	6	0
Isoform 2 of Transformation/transcription domain-associated protein	IPI00879277	2	0	0
Peroxiredoxin-2	IPI00027350 (+1)	8	6	4
Protein Plunc	IPI00009856 (+1)	10	6	4
Histatin-1	IPI00012024	4	4	12
Vimentin	IPI00418471 (+1)	6	2	8
Isoform 1 of Keratin, type I cytoskeletal 13	IPI00009866	6	0	4
Alpha-1-acid glycoprotein 2	IPI00020091	2	2	20
Putative uncharacterized protein DKFZp686I04196 (Fragment)	IPI00399007	8	4	4
Afamin	IPI00019943	0	0	8
Isoform 1 of Ribonuclease T2	IPI00414896 (+3)	6	2	0
Cathelicidin antimicrobial peptide precursor	IPI00292532	6	0	8
Coactosin-like protein	IPI00017704	2	0	4
13 kDa protein	IPI00829827	6	4	4
Isoform 1 of Alpha-actinin-1	IPI00013508 (+3)	4	0	8
Isoform 3 of Mesothelin	IPI00793649	4	4	8
Keratin, type I cytoskeletal 14	IPI00384444	2	8	8
Beta-2-glycoprotein 1	IPI00298828	2	0	4
L-lactate dehydrogenase B chain	IPI00219217	4	0	12
Uncharacterized protein	IPI01013441	2	0	4
Isoform 1 of Neutrophil gelatinase-associated lipocalin	IPI00299547 (+1)	8	6	0
Neutrophil collagenase	IPI00027846	0	0	20
Isoform 2 of Phosphoglucomutase-1	IPI00217872 (+2)	2	6	8
Isoform 1 of Vinculin	IPI00291175 (+1)	4	0	4
Small proline-rich protein 2E	IPI00386597	10	4	0
Isoform 1 of Brain acid soluble protein 1	IPI00299024	4	2	12
Heat shock-related 70 kDa protein 2	IPI00007702	2	2	0
Golgi membrane protein 1	IPI00171411 (+1)	4	4	12
Isoform 1 of Alpha-1B-glycoprotein	IPI00022895	8	2	8
Protein FAM3D	IPI00060143	4	0	8
Actin-related protein 2/3 complex subunit 3	IPI00005162	2	0	4
Ezrin	IPI00843975 (+1)	2	8	8
Aldehyde dehydrogenase, dimeric NADP-preferring	IPI00296183 (+1)	6	4	4
Basic salivary proline-rich protein 2	IPI00552432	6	4	4

Table S1: Complete list of identified proteins in HB, HC and Control (C) groups.

Isoform 2 of Poly(U)-specific endoribonuclease	IPI00006995 (+2)	6	0	0
Histone H2B type 2-E	IPI00003935 (+17)	4	0	4
Isoform 1 of Protein furry homolog-like	IPI00739940 (+1)	0	2	0
Hornerin	IPI00398625	2	2	0
cDNA FLJ78387	IPI00876888	4	2	0
calumenin isoform c precurosr	IPI00789155 (+1)	4	0	0
cDNA FLJ43795 fis, clone TESTI4000079	IPI00171456	2	0	4
Isoform 1 of Granulins	IPI00296713	6	4	0
Isoform 3 of Prominin-1	IPI00971071	2	4	0
Isoform 1 of Striated muscle preferentially expressed protein kinase	IPI00658151 (+1)	2	0	0
Talin-1	IPI00298994	6	0	4
Calmodulin-like protein 5	IPI00021536	4	6	4
33 kDa protein	IPI00967467	0	2	4
Coronin-1A	IPI00010133	4	2	8
Cathepsin B	IPI00295741 (+1)	0	2	4
cDNA FLJ59142, highly similar to Epididymal secretory protein E1	IPI00301579 (+2)	2	2	8
Isoform 1 of Electroneutral sodium bicarbonate exchanger 1	IPI00737057	0	0	4
Isoform Mitochondrial of Peroxiredoxin-5, mitochondrial	IPI00024915 (+2)	6	2	4
Isoform 1 of Liver carboxylesterase 1	IPI00010180 (+3)	2	0	0
Neutrophil defensin 1	IPI00005721 (+1)	2	2	16
Nicotinamide phosphoribosyltransferase	IPI00018873	4	0	12
Isoform Long of 14-3-3 protein beta/alpha	IPI00216318 (+1)	4	2	8
H/ACA ribonucleoprotein complex subunit 4 isoform 2	IPI00915274	0	0	4
Annexin A1	IPI00218918 (+1)	6	4	8
Transthyretin	IPI00022432 (+2)	4	2	0
Isoform 1 of Translation initiation factor eIF-2B subunit delta	IPI00005979 (+5)	6	0	4
Nucleoside diphosphate kinase	IPI00604590 (+1)	2	2	8
Heat shock 70 kDa protein 6	IPI00339269	6	2	0
Isoform 1 of Serpin B5	IPI00783625	2	2	8
Autophagy-related protein 2 homolog B	IPI00872410	2	0	0
Peptidoglycan recognition protein 1	IPI00021085	6	0	4
Uncharacterized protein	IPI00943181	4	4	4
Full-length cDNA 5-PRIME end of clone CS0DF001YB10 of Fetal brain of Homo sapiens (Fragment)	IPI01009227	2	2	0
Actin-related protein 2/3 complex subunit 4	IPI00554811 (+2)	6	2	0
Histone H2A type 1-D	IPI00255316	4	4	0
cDNA FLJ55673, highly similar to Complement factor B	IPI00019591 (+4)	4	2	8
Similar to Ig heavy chain V-I region HG3 precursor	IPI00739205	8	0	4
Galectin-3	IPI00465431	4	4	0
Similar to VH-3 family (VH26)D/J protein	IPI00985211	6	2	4
Uncharacterized protein	IPI00979039	0	0	4
Actin-related protein 3	IPI00028091	4	0	4
cDNA FLJ58687, highly similar to Tubulin alpha-4 chain	IPI00794663 (+1)	2	2	4
Histone H3.1	IPI00465070	2	2	4
Isoform 2 of 5'-3' exoribonuclease 1	IPI00657645	0	2	0

Table S1: Complete list of identified proteins in HB, HC and Control (C) groups.

Isoform Non-muscle of Myosin light polypeptide 6	IPI00335168 (+7)	2	2	12
Isoform 1 of Serpin B3	IPI00022204 (+1)	4	0	0
Isoform HMW of Kininogen-1	IPI00032328 (+3)	0	4	4
Flavin reductase	IPI00783862	0	8	0
Hemoglobin subunit gamma-1	IPI00220706	0	8	0
IGK@ protein	IPI00784865	8	2	0
Olfactomedin-4	IPI00022255	4	4	0
Secretoglobin family 3A member 1	IPI00066193	4	4	0
64 kDa protein	IPI00290078	0	0	12
Ig kappa chain V-III region VH (Fragment)	IPI00829834	4	2	4
Isoform 1 of Keratin, type II cytoskeletal 78	IPI00477227	0	4	0
Ubiquitin-like modifier-activating enzyme 1	IPI00645078	2	0	4
Isoform 2 of Neutral alpha-glucosidase AB	IPI00011454 (+1)	0	2	0
Nucleotide exchange factor SIL1	IPI00296197	2	0	0
Proteasome activator complex subunit 1	IPI00479722	2	4	0
Synaptic vesicle membrane protein VAT-1 homolog	IPI00156689	6	0	8
Ras GTPase-activating-like protein IQGAP1	IPI00009342	0	0	4
Involucrin	IPI00011692 (+1)	4	4	0
Corticosteroid-binding globulin	IPI00027482	2	4	0
Inositol polyphosphate 1-phosphatase	IPI00027139 (+2)	4	0	0
NAD(P) transhydrogenase, mitochondrial	IPI00337541 (+1)	0	0	4
Isoform RF1/RF2 of Retrotransposon-derived protein PEG10	IPI00022095 (+2)	0	0	4
Collagen alpha-4(IV) chain	IPI00478572 (+1)	0	2	0
Isoform 2 of Actin-binding LIM protein 1	IPI00456617 (+2)	2	0	0
cDNA FLJ59211, highly similar to Glucosidase 2 subunit beta	IPI00026154 (+1)	0	6	0
cDNA FLJ54977, weakly similar to Proline-rich protein 4	IPI00856018	4	2	0
38 kDa protein	IPI00872245	2	0	0
Isoform A of Golgi SNAP receptor complex member 2	IPI00023135 (+3)	2	0	0
C-myc promoter-binding protein	IPI00396063 (+1)	2	0	0
Similar to Hepatitis B virus receptor binding protein	IPI00983475	0	0	4
Malate dehydrogenase, mitochondrial	IPI00291006	4	1	0
Isoform 2 of Prostatic acid phosphatase	IPI00289983 (+2)	4	2	0
Resistin	IPI00006988	2	0	4
Isoform 1 of Apoptosis-associated speck-like protein containing a CARD	IPI00001699 (+2)	0	2	4
Isoform 1 of Arginase-1	IPI00291560 (+1)	2	2	0
Isoform 2 of Protein CASC5	IPI00170766 (+1)	0	2	0
Tubulin alpha-1A chain	IPI00180675 (+4)	0	2	4
28S ribosomal protein S22, mitochondrial	IPI00013146 (+2)	0	2	0
Chloride intracellular channel protein 1	IPI00010896	0	2	0
Adenylosuccinate synthetase isozyme 2	IPI00026833	0	0	8
Isoform 2 of Neural cell adhesion molecule L1-like protein	IPI00299059	2	0	0
Pre-mRNA-processing factor 6	IPI00305068 (+1)	0	2	0
Carbonic anhydrase 2	IPI00218414	2	6	0
Hemoglobin Lepore-Baltimore (Fragment)	IPI00829896	2	6	0

Table S1: Complete list of identified proteins in HB, HC and Control (C) groups.

Isocitrate dehydrogenase [NADP] cytoplasmic	IPI00027223	0	2	4
Isoform 1 of Oxysterol-binding protein-related protein 2	IPI00014137	2	0	0
Isoform 2 of Protein Wnt-2b	IPI00004121 (+2)	2	0	0
Enamelin	IPI00016685	2	0	0
Cystatin-A	IPI00032325	4	0	0
Isoform 2 of Keratin, type II cytoskeletal 80	IPI00431749 (+1)	0	4	0
Furin	IPI00018387	2	2	0
perilipin-3 isoform 3	IPI00106668 (+2)	0	0	4
Carboxypeptidase D	IPI00027078 (+2)	2	0	4
Acylamino-acid-releasing enzyme	IPI00337741 (+1)	0	0	4
ADP-ribosylation factor 1	IPI00215914 (+2)	0	0	8
Ig lambda chain V-IV region MOL	IPI00386576	4	0	0
Inter-alpha (Globulin) inhibitor H2, isoform CRA_a	IPI00305461 (+2)	0	1	0
Protein FAM49B	IPI00303318	0	0	12
Isoform 1 of Ermin	IPI00006746 (+1)	2	2	0
Isoform 1 of WD repeat-containing protein 1	IPI00746165	2	0	0
Uncharacterized protein	IPI00922694	0	2	0
Ig lambda chain V region 4A	IPI00022890	1	0	0
Actin-related protein 2	IPI00005159	0	0	8
Extracellular glycoprotein lacritin	IPI00020487	0	3	0
Isoform 1 of Glycerophosphodiester phosphodiesterase domain-containing protein 4	IPI00179751 (+1)	2	0	0
Uncharacterized protein	IPI00472782 (+1)	0	4	0
Isoform 1 of Holliday junction recognition protein	IPI00301465 (+2)	2	0	0
Apolipoprotein A-II	IPI00021854	0	2	0
Isoform 1 of Protein arginine N-methyltransferase 3	IPI00401321 (+2)	0	0	4
DNA-(apurinic or apyrimidinic site) lyase 2	IPI00083281	2	2	0
Isoform 1 of 45 kDa calcium-binding protein	IPI00106646	2	0	0
Isoform 1 of RNA-binding protein 5	IPI00005036 (+1)	2	0	0
Translin	IPI00018768 (+2)	0	4	0
Isoform 1 of Bromodomain-containing protein 8	IPI00019226	4	0	0
Mammaglobin-B	IPI00026126	0	4	0
Serum paraoxonase/arylesterase 1	IPI00218732 (+1)	0	4	0
Superoxide dismutase [Cu-Zn]	IPI00218733 (+1)	0	0	4
Inter-alpha-trypsin inhibitor heavy chain H1	IPI00292530 (+1)	4	0	0
Isoform 2 of Protein disulfide-isomerase A6	IPI00299571 (+4)	7	0	0
Apolipoprotein A-IV	IPI00304273	0	2	0
Keratin, type I cytoskeletal 19	IPI00479145	0	0	4
51 kDa protein	IPI00646774	0	2	0
Uncharacterized protein	IPI00789324	0	2	0
13 kDa protein	IPI00829845	0	0	4
Isoform 2 of Transmembrane protein 132D	IPI00845291	0	2	0
Alcohol dehydrogenase class 4 mu/sigma chain	IPI00872487 (+2)	4	0	0
Tissue alpha-L-fucosidase	IPI00843910	2	0	0
Similar to AUTS2-like protein	IPI00973550	2	0	0

Table S1: Complete list of identified proteins in HB, HC and Control (C) groups.

Leucine-rich repeat and WD repeat-containing protein KIAA1239	IPI00166979	2	0	0
Vitronectin	IPI00298971 (+1)	0	0	4
Isoform 1 of Origin recognition complex subunit 3	IPI00294402 (+3)	2	0	0
Testis-specific serine/threonine-protein kinase 1	IPI00012465	2	0	0
Origin recognition complex subunit 6	IPI00001641	2	0	0
Isoform 1 of HBS1-like protein	IPI00009070 (+6)	2	0	0
ADP-ribosylation factor-like protein 8A	IPI00060031	2	0	0
Isoform 1 of Splicing regulatory glutamine/lysine-rich protein 1	IPI00103497 (+1)	2	0	0
Protein notum homolog	IPI00465159 (+1)	2	0	0
